# A novel therapeutic strategy against gastric carcinoma: artemisia sieberi-derived biogenic zno nanoparticles inducing intrinsic apoptosis and suppressing AGS cell proliferation

**DOI:** 10.1038/s41598-026-43373-3

**Published:** 2026-06-19

**Authors:** Nilofar Akbarzadeh, Mohammadreza Pourmohammad, Homa Mahmoudzadeh, Maryam Tehranipour

**Affiliations:** 1 Department of Biology, M.Sc., Islamic Azad University, Mashhad, Iran; 2 Department of Parasitology and Mycology, School of Allied Medical Sciences, Ilam University of Medical Sciences, Ilam, Iran

**Keywords:** Gastric cancer, Zinc oxide nanoparticles, Artemisia sieberi, Apoptosis, Cell migration, Biochemistry, Biotechnology, Cancer, Cell biology, Drug discovery

## Abstract

Gastric carcinoma remains a major cause of cancer-related mortality worldwide, underscoring the need for alternative therapeutic strategies. Zinc oxide nanoparticles (ZnO NPs) have attracted attention in cancer research due to their ability to generate reactive oxygen species (ROS), relative cost-effectiveness, and suitability for green synthesis approaches. In this study, ZnO NPs were synthesized via an eco-friendly biogenic method using *Artemisia sieberi*, and their anticancer-related effects were evaluated in vitro. ZnO nanoparticles were synthesized using an aqueous extract of *Artemisia sieberi* and characterized by FE-SEM, DLS, EDS, and XRD. Their biological effects were investigated in human gastric adenocarcinoma (AGS) cells and normal human embryonic kidney (HEK-293) cells. Cytotoxicity was assessed using the MTT assay, apoptosis by flow cytometry, cell migration by a wound healing assay, and gene expression by qRT-PCR. Statistical analysis was performed using one-way ANOVA, with *p* < 0.05 considered significant. Physicochemical characterization confirmed the formation of spherical to polyhedral ZnO nanoparticles with sizes ranging from 37 to 122 nm. The nanoparticles exhibited concentration-dependent cytotoxicity in AGS cells, with an IC₅₀ of approximately 480 µg/mL, compared to an IC₅₀ of approximately 1250 µg/mL in HEK-293 cells, corresponding to a modest selectivity index (~ 2.6). Flow cytometric analysis demonstrated increased apoptosis in AGS cells, with up to 61.6% apoptotic cells observed at the highest concentration. In addition, ZnO NPs significantly inhibited cancer cell migration. Molecular analysis revealed significant upregulation of *p53*, *Caspase-9*, and *Caspase-3*, suggesting activation of the intrinsic mitochondrial apoptotic pathway. The findings indicate that *Artemisia sieberi*-derived ZnO nanoparticles can induce apoptosis and inhibit migration in gastric cancer cells in vitro, potentially through a p53-mediated mitochondrial mechanism. Although the observed selectivity toward cancer cells was limited, these results support the preliminary potential of biogenically synthesized ZnO nanoparticles as candidates for further investigation. Additional studies, including in vivo models and broader mechanistic analyses, are required to clarify their therapeutic relevance.

## Introduction

Gastric carcinoma remains one of the most prevalent malignancies worldwide and is a leading cause of cancer-related mortality^1^. Despite significant advances in diagnostic and therapeutic strategies, the prognosis of patients with gastric cancer remains poor, largely due to late-stage diagnosis, frequent recurrence, and the development of chemoresistance^2^. These challenges highlight the urgent need for novel therapeutic approaches that offer improved efficacy with reduced systemic toxicity^3^.

Nanotechnology has emerged as a promising tool in cancer research, providing new opportunities for targeted diagnosis and therapy^4^. Among various nanomaterials, zinc oxide nanoparticles (ZnO NPs) have attracted attention because of their ability to induce oxidative stress, disrupt mitochondrial function, and activate apoptotic signaling pathways in cancer cells^5^. However, the biological effects of ZnO NPs are strongly influenced by factors such as particle size, surface characteristics, concentration, and cellular context. Consequently, their biocompatibility and cytotoxicity are dose- and system-dependent rather than inherently benign, necessitating careful evaluation in each experimental model^6^.

In recent years, green synthesis of nanoparticles using plant extracts has been proposed as an environmentally friendly and cost-effective alternative to conventional chemical and physical methods^7^. In these phytogenic approaches, plant-derived biomolecules act as natural reducing and stabilizing agents, potentially modifying nanoparticle surface properties and influencing biological interactions^8^. Nevertheless, the biological activity of such nanoparticles must be experimentally validated rather than assumed.

*Artemisia sieberi* is a medicinal plant traditionally used in the Middle East and Central Asia and is known for its antimicrobial, antioxidant, and anticancer-related properties^9^. Its phytochemical constituents, including flavonoids, terpenoids, and phenolic compounds, have been reported to modulate oxidative stress and apoptosis-related pathways in various cancer models^10^. The incorporation of *A. sieberi* extract into nanoparticle synthesis may therefore influence the physicochemical and biological properties of the resulting ZnO NPs^11^.

Despite increasing interest in plant-mediated ZnO nanoparticles, there are currently no reports describing the anticancer efficacy or underlying molecular mechanisms of *Artemisia sieberi*–derived ZnO nanoparticles in human gastric carcinoma cells. In particular, their effects on apoptosis-related gene expression in AGS gastric cancer cells remain unexplored.

In the present study, ZnO nanoparticles were synthesized using *Artemisia sieberi* extract and their cytotoxic and pro-apoptotic effects were evaluated in AGS human gastric carcinoma cells. In parallel, their biocompatibility was assessed in normal HEK-293 human embryonic kidney cells. Furthermore, alterations in the expression of key apoptosis-related genes, including *p53*, *caspase-3*, and *caspase-9*, were analyzed to provide mechanistic insight into the potential of these biogenic ZnO nanoparticles as selective anticancer agents.

## Materials and methods

This study was carried out at the Applied Biology Research Center, Islamic Azad University, Mashhad Branch, Mashhad, Iran, during the first half of 2024. Ethical approval for the research was obtained from the Ethics Committee of the Islamic Azad University, Mashhad Medical Sciences Branch (Approval Code: IR.IAU.MSHD.REC.1403.015). All experimental protocols were conducted in full compliance with institutional regulations and internationally accepted ethical standards for biomedical research.

### Cell Culture

The human gastric adenocarcinoma cell line AGS and the human embryonic kidney fibroblast cell line HEK-293 were procured from the Pasteur Institute of Iran. Both cell lines were cultured under sterile conditions to maintain optimal viability and proliferation. AGS cells were maintained in RPMI-1640 medium supplemented with 10% fetal bovine serum (FBS) and 1% antibiotic solution, while HEK-293 cells were propagated in DMEM containing 10% FBS and 1% antibiotic solution. Both cell lines were incubated at 37 °C in a humidified atmosphere with 5% CO₂. Upon reaching 70–80% confluence, cell morphology and adherence were verified microscopically. Cells were detached using trypsin for 5 min, neutralized with fresh medium at twice the volume of trypsin, centrifuged at 3600 rpm for 4 min, and resuspended in fresh medium. Cell viability was assessed using the trypan blue exclusion assay, and viable cells were counted with a Neubauer hemocytometer. For experiments, cells were seeded at a density of 5 × 10³ cells per well in 96-well plates, a standard and reproducible value, and allowed to adhere for 24 h at 37 °C, 90% relative humidity, and 5% CO₂. All experiments were performed in triplicate (three independent biological replicates) with three technical replicates per sample, ensuring reproducibility. HEK-293 cells were used as a non-cancerous control to evaluate cytotoxicity in normal human cells; although non-malignant gastric epithelial cells (e.g., GES-1) could be used in future studies, HEK-293 provides a well-characterized and widely accepted baseline for comparison^12^.

### Green synthesis and characterization of ZnO NPs using artemisia vulgaris extract

The medicinal plant *Artemisia sieberi* (Herbarium code: ASM-2026-0157-MH**)** was collected from the greenhouse of Ferdowsi University of Mashhad, and the dried leaves were ground into a fine powder for preparation of an aqueous extract. Briefly, 15 g of the powdered leaves were mixed with 100 mL of distilled water and heated for 5 min, then filtered to obtain the clear extract. For nanoparticle synthesis, 1.83 g of zinc acetate was dissolved in 50 mL of distilled water, and 1 mL of this solution was added to 10 mL of the plant extract under constant stirring for 15 min to facilitate the formation of ZnO nanoparticles. The resulting nanoparticles were collected by centrifugation and washed three times with deionized water to remove impurities. To differentiate the effects of phytochemical capping, chemically synthesized ZnO nanoparticles were prepared under similar conditions without the plant extract as controls. The nanoparticles were characterized using X-ray diffraction (XRD), transmission electron microscopy (TEM), field-emission scanning electron microscopy (FESEM), energy-dispersive X-ray spectroscopy (EDS), dynamic light scattering (DLS), and zeta potential measurements to assess crystal structure, morphology, particle size, and colloidal stability^13^.

### Cytotoxicity of ZnO nanoparticles assessed by MTT assay

The cytotoxic effects of the synthesized ZnO NPs were evaluated using the MTT (3-(4,5-dimethylthiazol-2-yl)-2,5-diphenyltetrazolium bromide) assay. Human gastric adenocarcinoma (AGS) and human embryonic kidney (HEK-293) cells were seeded into 96-well plates and treated with ZnO NPs at concentrations optimized around the expected IC₅₀ values. A stock solution of MTT was prepared at 5 mg/mL in phosphate-buffered saline (PBS), and 10 µL of this solution was added to each well. Following incubation at 37 °C for 3–4 h in the dark, metabolically active cells reduced the yellow tetrazolium salt into insoluble purple formazan crystals via mitochondrial dehydrogenases. The culture medium was then carefully aspirated, and 80 µL of dimethyl sulfoxide (DMSO) was added to solubilize the formazan crystals. The absorbance was measured at 560–570 nm using a microplate spectrophotometer (BioTek, USA)^14^.

### DAPI staining for nuclear morphological analysis

Nuclear morphology and apoptotic changes in AGS cells were analyzed using DAPI (4′,6-diamidino-2-phenylindole) staining. Cells were seeded onto sterile coverslips in 24-well plates and allowed to adhere. Upon reaching the desired confluence, cells were treated with varying concentrations of the test compound for 24 h, with untreated cells serving as a control group. At the conclusion of the incubation period, the culture medium was aspirated, and cells were fixed with methanol for 10 min at room temperature. After fixation, the methanol was removed, and 300 µL of DAPI staining solution was added to each well. Following a 10-minute incubation in the dark, the cells were gently washed to remove excess dye and subsequently examined using an inverted fluorescence microscope. DAPI, a blue-fluorescent DNA-binding dye with a strong affinity for adenine-thymine (A-T) rich regions, allowed for the visualization of nuclear alterations. Apoptotic cells were identified by the presence of condensed or fragmented chromatin, whereas viable cells displayed normal, uniformly stained nuclei. This assay provided a morphological assessment of nuclear damage and apoptosis induction in treated versus control groups^15^.

### Apoptosis assessment using annexin V/Propidium Iodide staining

The induction of apoptosis in AGS gastric cancer cells following treatment with various concentrations of Artemisia sieberi extract was assessed using Annexin V/Propidium Iodide (PI) staining with a commercial apoptosis detection kit (Abcam, UK). Briefly, approximately 1 × 10⁶ cells were harvested and centrifuged at 1500 rpm for 5 min, and the supernatant was discarded. The cell pellet was washed once with cold phosphate-buffered saline (PBS) and centrifuged again under the same conditions. Cells were then resuspended in 500 µL of 1× Annexin V binding buffer. The suspension was stained by adding 10 µL of Annexin V-FITC and 5 µL of PI, followed by incubation in the dark at room temperature for 15 min. Flow cytometry was performed using a BD FACSCalibur cytometer, applying a forward and side scatter (FSC/SSC) gating strategy to exclude debris and select the cell population of interest. Single-stained controls were used for fluorescence compensation, and at least 10,000 events per sample were acquired to ensure sufficient statistical representation^16^.

### Wound healing assay for assessment of cell migration

The anti-migratory effect of the compound was evaluated in AGS gastric carcinoma cells using a wound healing assay. Briefly, 1 × 104 cells per well were seeded in 6-well plates and cultured to approximately 80% confluence. A linear scratch was created across the diameter of each well using a sterile pipette tip to simulate a wound. Following the scratch, cells were treated with predetermined effective concentrations of the compound and incubated for 24 h. Cell migration into the wound area was monitored using an inverted microscope, and images were captured at both 0 and 24 h post-treatment. The extent of wound closure and cellular motility was qualitatively compared between treated and control groups to assess the compound’s inhibitory effect on AGS cell migration^17^.

### RNA extraction and cDNA synthesis

Total RNA was extracted from cultured cells using the Pars Tous RNA extraction kit according to the manufacturer’s protocol. Adherent cells were harvested, lysed, and purified through a series of washes and centrifugations. The concentration and purity of the extracted RNA were assessed by spectrophotometry. cDNA was synthesized using the Pars Tous cDNA synthesis kit in a thermocycler under the following program: 25 °C for 10 min, 47 °C for 60 min, and 85 °C for 5 min to inactivate the reverse transcriptase. The synthesized cDNA was stored at − 20 °C and used for subsequent real-time PCR analysis. GAPDH was used as the housekeeping gene for normalization in all qRT-PCR experiments^18^.

### Real-time PCR analysis

Real-time quantitative PCR (RT-qPCR) was performed to quantify the relative expression levels of apoptosis-related genes in human gastric adenocarcinoma (AGS) cells. Synthesized cDNA from treated and untreated samples served as the template for amplification using a Bio-Rad CFX96 Real-Time PCR Detection System. Specific primers for the target genes (Caspase-3, Caspase-9, and p53) are listed in Table 1, and GAPDH was used as the housekeeping gene for normalization^19^. Relative gene expression was calculated using the 2⁻ΔΔCt method. For statistical analysis, data from three independent biological replicates were analyzed. Differences between groups were assessed using one-way ANOVA, considering treatment concentration and time as factors, with assumptions of normality and homogeneity of variances checked prior to analysis.

**Table 1:** Primer sequences and gene details for quantitative real-time PCR analysis.

Gene Symbol	Gene Name	Primers (5ʹ → 3ʹ)
**GAPDH**	Glyceraldehyde-3-phosphate dehydrogenase	Forward: 5’-TGA CTT CAA CAG CGA CAC C-3’
		Reverse: 5’-TTG CTG TAG CCA AAT TCG TT-3’
**P53**	Tumor suppressor protein	Forward: 5’-TTG CCG TCC CAA GCA ATG GA-3’
		Reverse: 5’-TCT GGG AAG GGA CAG AAG ATG-3’
**Caspase3**	Cysteine-aspartic acid protease 3	Forward: 5’-AGA CAG ACA GTG GTG TTG ATG-3’
		Reverse: 5’-GTT CAT CCA GTC GCT TTG TGC-3’
**Caspase9**	Cysteine-aspartic acid protease 9	Forward: 5’-CCA GAG ATT CGC AAA CCA GAG-3’
		Reverse: 5’-CAA TGT GAA CTT CTG CCG TGA-3’

### Statistical analysis

All quantitative data were analyzed from a completely randomized experimental design with three independent biological replicates, each performed in triplicate. Statistical computations were performed using GraphPad Prism (Version 9.0). One-way ANOVA was applied with treatment concentration and time as factors, and assumptions of normality and homogeneity of variances were verified prior to analysis. Post hoc comparisons were conducted using Tukey’s HSD test, and a p-value of < 0.05 was considered statistically significant.

## Result

### Morphological analysis

**Fig. 1:**
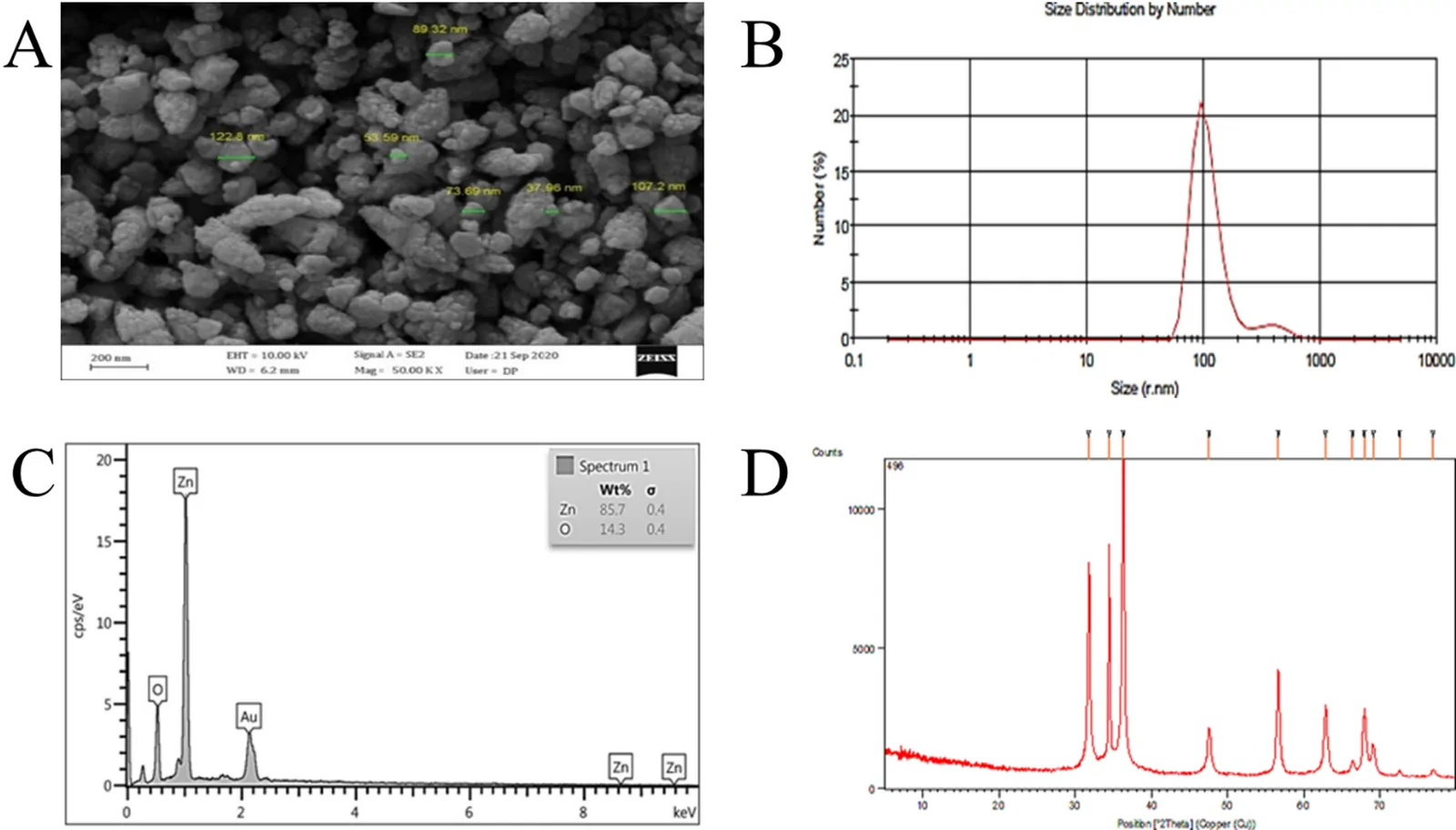
Characterization of ZnO nanoparticles synthesized using *Artemisia sieberi* extract, showing (**A**) FE-SEM image of nanoparticle morphology, (**B**) DLS size distribution, (**C**) EDS spectrum confirming elemental composition, and (**D**) XRD pattern indicating crystalline structure.

The surface morphology of the synthesized ZnO nanoparticles was meticulously investigated using field-emission scanning electron microscopy (FE-SEM), with representative images presented in Fig. 1A. The nanoparticles predominantly exhibited a range of morphologies from spherical to polyhedral, with sizes spanning 37 to 122 nm. Comparison with DLS measurements revealed larger hydrodynamic diameters ranging from 95 to 105 nm, which is expected due to solvation effects and possible interparticle interactions in suspension. The polydispersity index (PDI) obtained from DLS was 0.12, indicating a moderately polydisperse system, which may influence colloidal behavior and functional performance. This observed size heterogeneity is characteristic of a biogenic synthesis pathway, which typically involves heterogeneous nucleation and growth processes. At higher nanoparticle concentrations (1 mg/mL), some degree of aggregation was apparent, likely driven by van der Waals interactions and high particle proximity; this phenomenon can be visualized using brightfield microscopy (Fig. 1A), highlighting the importance of dispersion conditions for applications. Such aggregation can affect the effective surface area and, consequently, the catalytic or biological activity of the nanoparticles. The high magnification (50,000×) employed during FE-SEM imaging provided detailed insight into the microstructural features, confirming the nanoscale dimensions and the general morphological uniformity of the material, despite the inherent polydispersity.

### Particle size distribution

The particle size distribution of the synthesized ZnO nanoparticles was determined via DLS, as shown in Fig. 1B. The DLS measurements revealed a narrow, monomodal distribution centered at approximately 100 nm, which is slightly larger than the 37–122 nm range observed by FE-SEM. This difference arises because DLS measures the hydrodynamic diameter, including solvation layers and weak interparticle interactions. The low polydispersity index (PDI = 0.12) and the sharp peak in the distribution curve indicate relatively uniform particle formation with minimal aggregation in suspension. Such moderate polydispersity is beneficial for biological applications, as it can enhance cellular uptake, reduce cytotoxicity, and ensure reproducible interactions with biomolecules.

### Elemental composition analysis

The elemental composition of the synthesized ZnO nanoparticles was rigorously assessed using energy-dispersive X-ray spectroscopy (EDS), with the corresponding spectrum displayed in Fig. 1C. The analysis revealed dominant peaks for zinc (Zn) and oxygen (O), with calculated weight percentages of 85.7% and 14.3%, respectively. A minor peak corresponding to gold (Au) was also detected, which is attributed to the sputter-coating process used to enhance sample conductivity for FE-SEM analysis. The absence of other impurity elements confirms the high purity of the synthesized ZnO nanoparticles, and these findings are in complete agreement with the expected chemical composition of the target nanomaterial.

### X-ray diffraction (XRD) analysis

The crystallographic properties of the synthesized ZnO nanoparticles were examined via XRD, as shown in Fig. 1D. The XRD pattern confirmed that the nanoparticles possess a single-phase crystalline structure, with no discernible peaks corresponding to impurities. The observed diffraction peaks at 2θ values of 11.5∘, 16.9∘, 29.7∘, 32.3∘, 33.5∘, 36∘, 37.8∘, 40∘, and 45∘ were successfully indexed to the (100), (200), (101), (102), (110), (103), (112), (201), and (400) crystallographic planes of polyhedral ZnO nanoparticles, respectively. The sharpness and high intensity of these peaks are indicative of high crystallinity and phase purity, thereby validating the presence of well-defined crystal domains.

### Selective cytotoxicity: an MTT assay analysis

**Fig. 2:**
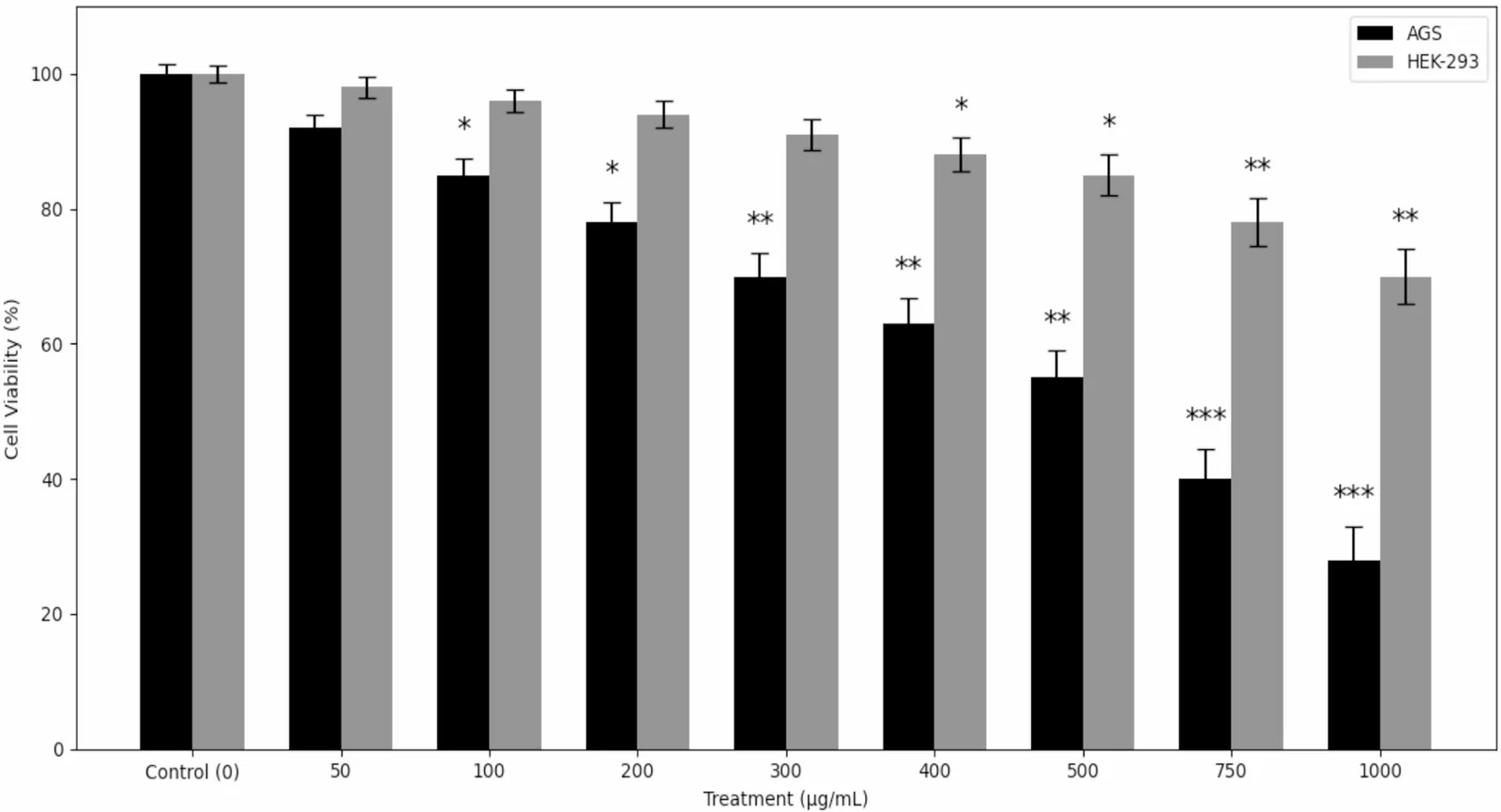
Dose-dependent effects of the compound on AGS and HEK-293 cell viability (0–1000 µg/mL). Data are mean ± SD (*n* = 3). *p < 0.05, **p < 0.01, ***p < 0.001 vs. control.

The cytotoxic effects of the synthesized compound were evaluated on AGS gastric cancer cells and normal HEK-293 cells over a concentration range of 0–1000 µg/mL (Fig. 2). AGS cells showed a dose-dependent decrease in viability, with a statistically significant reduction at 100 µg/mL (*p* = 0.032) and a viability of 28% at 1000 µg/mL (*p* < 0.001). The IC₅₀ for AGS cells was approximately 480 µg/mL. In contrast, HEK-293 cells exhibited significant viability reduction only at concentrations above 400 µg/mL (*p* = 0.041), with viability remaining around 70% at 1000 µg/mL, corresponding to an IC₅₀ of ~ 1250 µg/mL. The selectivity of the compound for AGS cells versus HEK-293 was ~ 2.6-fold. Additional concentrations near the IC₅₀ confirmed the dose-dependent response.

### DAPI staining analysis: assessment of apoptotic nuclear morphology

**Fig. 3:**
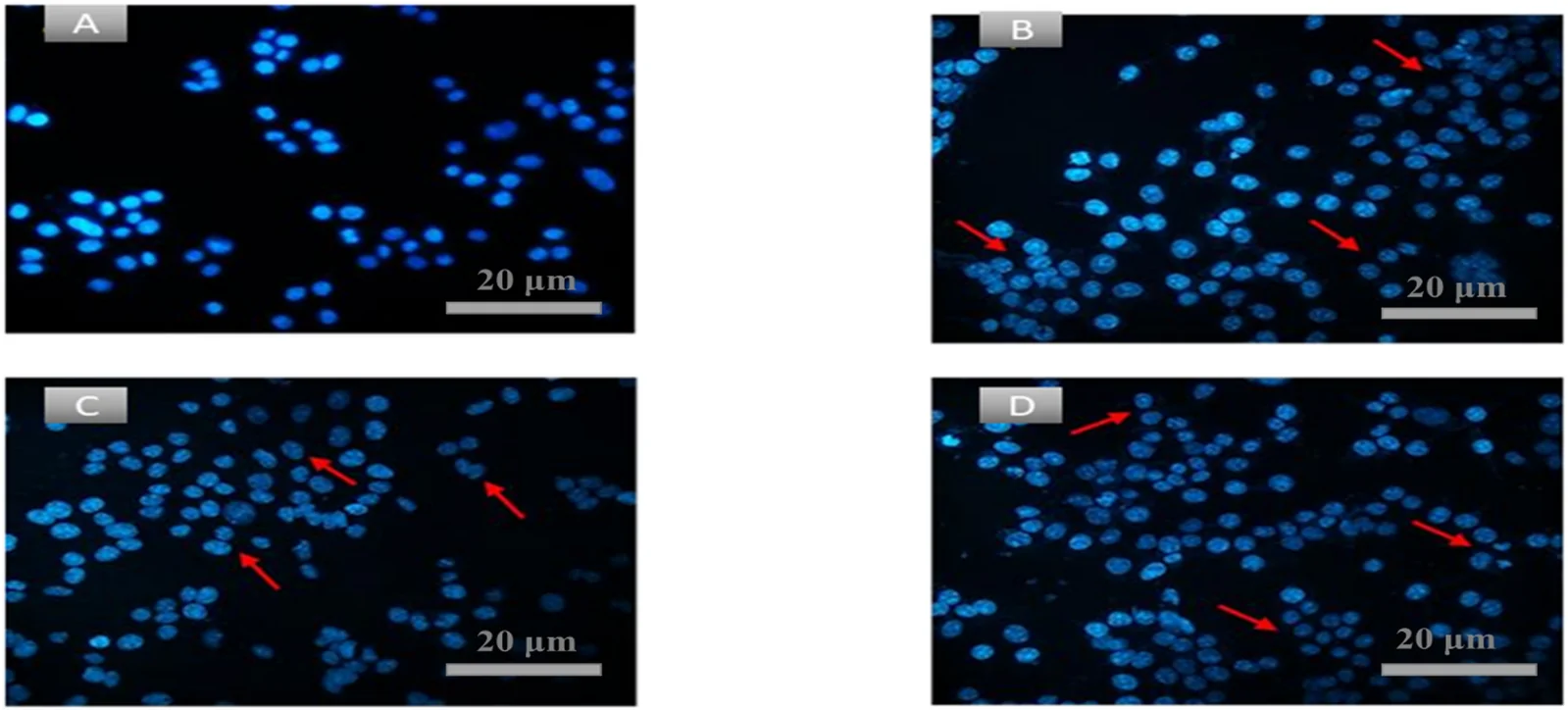
DAPI-stained nuclei of AGS cells following treatment with the compound at 0 (control, **A**), 50 (**B**), 480 (**C**), and 1000 µg/mL (**D**). Red arrows indicate nuclear condensation and fragmentation, indicative of apoptosis.

The apoptotic effects of the compound on AGS gastric cancer cells were assessed by evaluating nuclear morphology using DAPI staining (Fig. 3). Untreated control cells exhibited large, round nuclei with uniform chromatin, indicative of healthy cells. Upon treatment, dose-dependent apoptotic changes were observed. At 50 µg/mL, mild nuclear condensation and incipient fragmentation were detected. At the estimated IC₅₀ concentration (480 µg/mL), a substantial increase in cells with condensed and fragmented nuclei was observed. The highest concentration (1000 µg/mL) induced extensive chromatin condensation and nuclear fragmentation in the majority of cells. These results confirm a clear dose-dependent induction of apoptosis in AGS cells as evidenced by nuclear morphological changes. To avoid confounding effects from cytotoxicity, sub-lethal concentrations were used for assessing apoptotic morphology.

### Quantitative assessment of apoptosis by flow cytometry

**Fig. 4:**
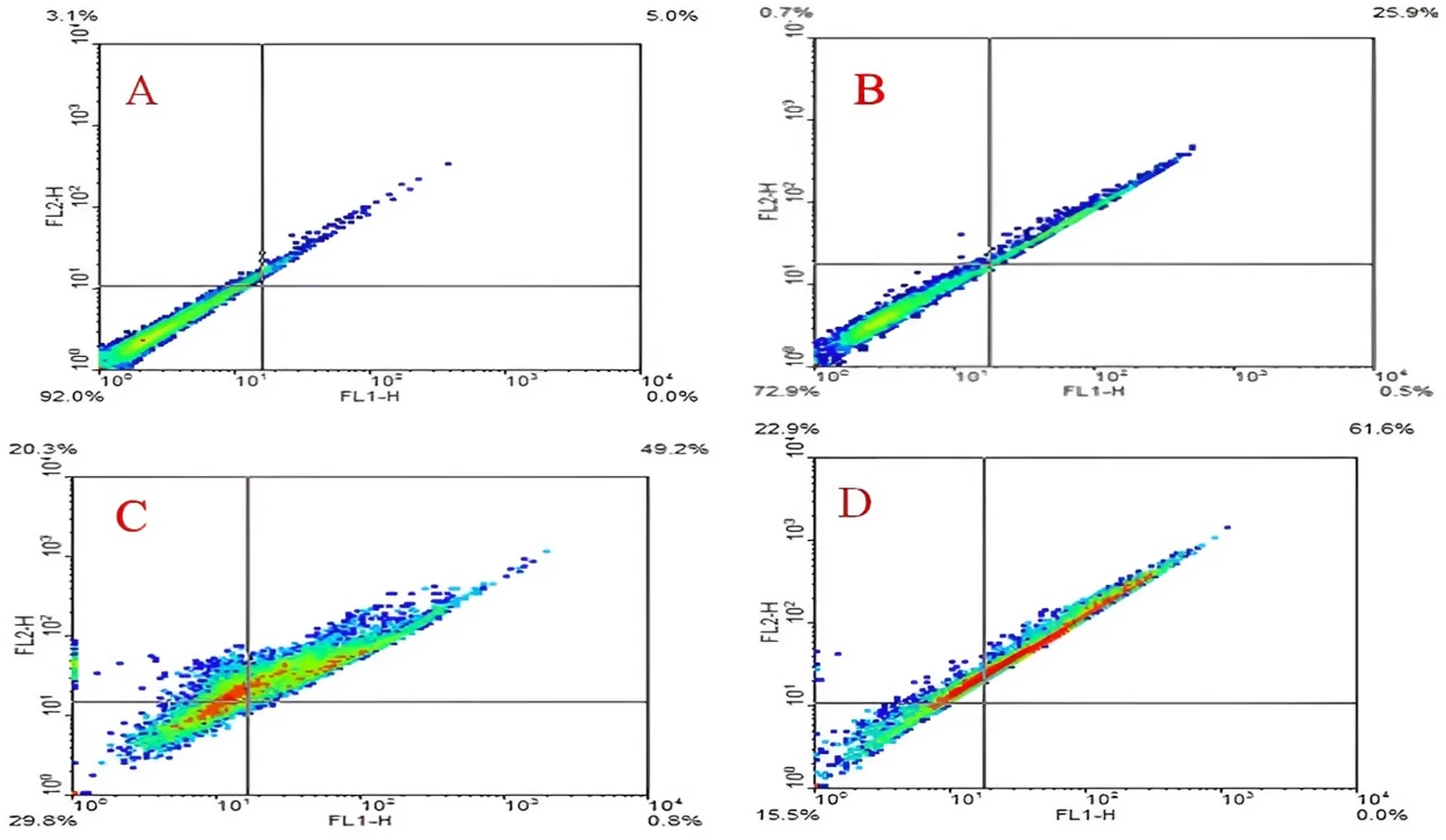
Flow cytometry analysis of apoptosis in AGS cells treated with biogenic ZnO nanoparticles at 0 (control, **A**), 50 (**B**), 480 (**C**), and 1000 µg/mL (**D**).

Flow cytometry analysis revealed a dose-dependent induction of apoptosis in AGS gastric cancer cells following treatment with biogenic ZnO NPs derived from *Artemisia sieberi* (Fig. 4). In the control group (Fig. 4A), 92% of cells were in the lower-left quadrant, representing healthy cells with low baseline fluorescence in both FL1-H and FL2-H channels. Treatment with 50 µg/mL ZnO NPs (Fig. 4B) increased the proportion of double-positive cells in the upper-right quadrant to 25.9%, indicative of early apoptotic activation. At 480 µg/mL (Fig. 4C), 49.2% of cells were double-positive, and at 1000 µg/mL (Fig. 4D), apoptosis peaked at 61.6%. These results demonstrate a progressive, dose-dependent apoptotic response. To ensure reproducibility, the analysis was performed in triplicate, with consistent gating parameters across all samples.

### Cell migration assay

**Fig. 5:**
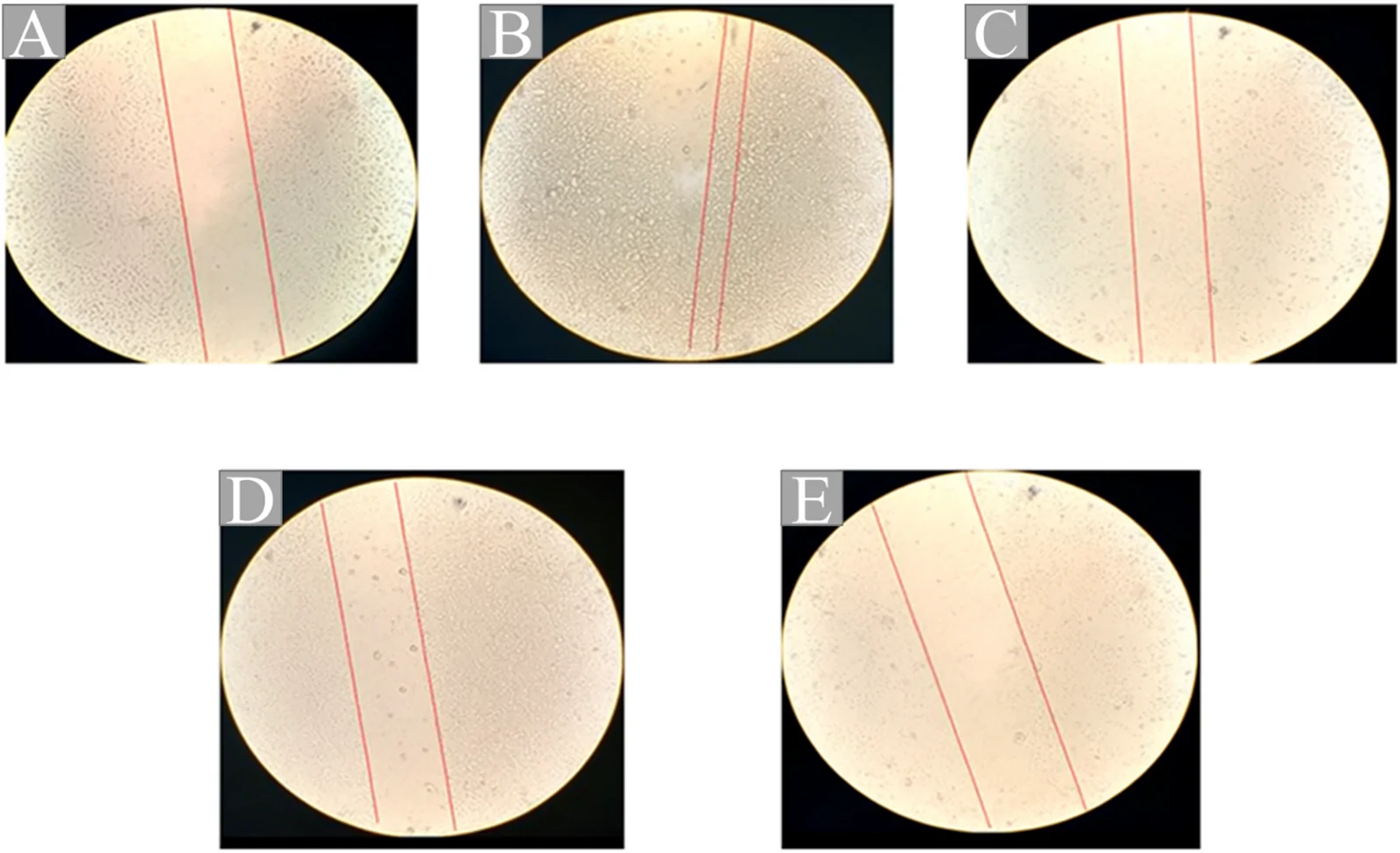
Representative wound healing assay images of AGS cells treated with biogenic ZnO nanoparticles. (**A**) scratch area at 0 h, (**B**) untreated control after 24 h, and treated groups with (**C**) 50 µg/mL, (**D**) 480 µg/mL, and (**E**) 1000 µg/mL. Images captured after 24 h demonstrate a dose-dependent inhibition of cell migration. Magnification: ×100.

A wound healing assay was used to evaluate the anti-migratory potential of biogenic ZnO NPs derived from *Artemisia sieberi* on AGS gastric cancer cells (Fig. 5). At 0 h, a uniform scratch gap was observed across all groups (Fig. 5A). After 24 h, untreated control cells showed near-complete closure of the wound (Fig. 5B). Cells treated with 50 µg/mL (Fig. 5C) and 480 µg/mL (Fig. 5D) exhibited partial wound closure, whereas 1000 µg/mL (Fig. 8E) resulted in pronounced inhibition, leaving the scratch gap largely open. These results indicate a concentration-dependent suppression of AGS cell migration by the ZnO NPs. It should be noted that wound closure was assessed at a single time point, and migration versus proliferation was not distinguished, which limits kinetic interpretation.

### ZnO nanoparticles induce apoptosis: A qRT-PCR analysis

**Fig. 6:**
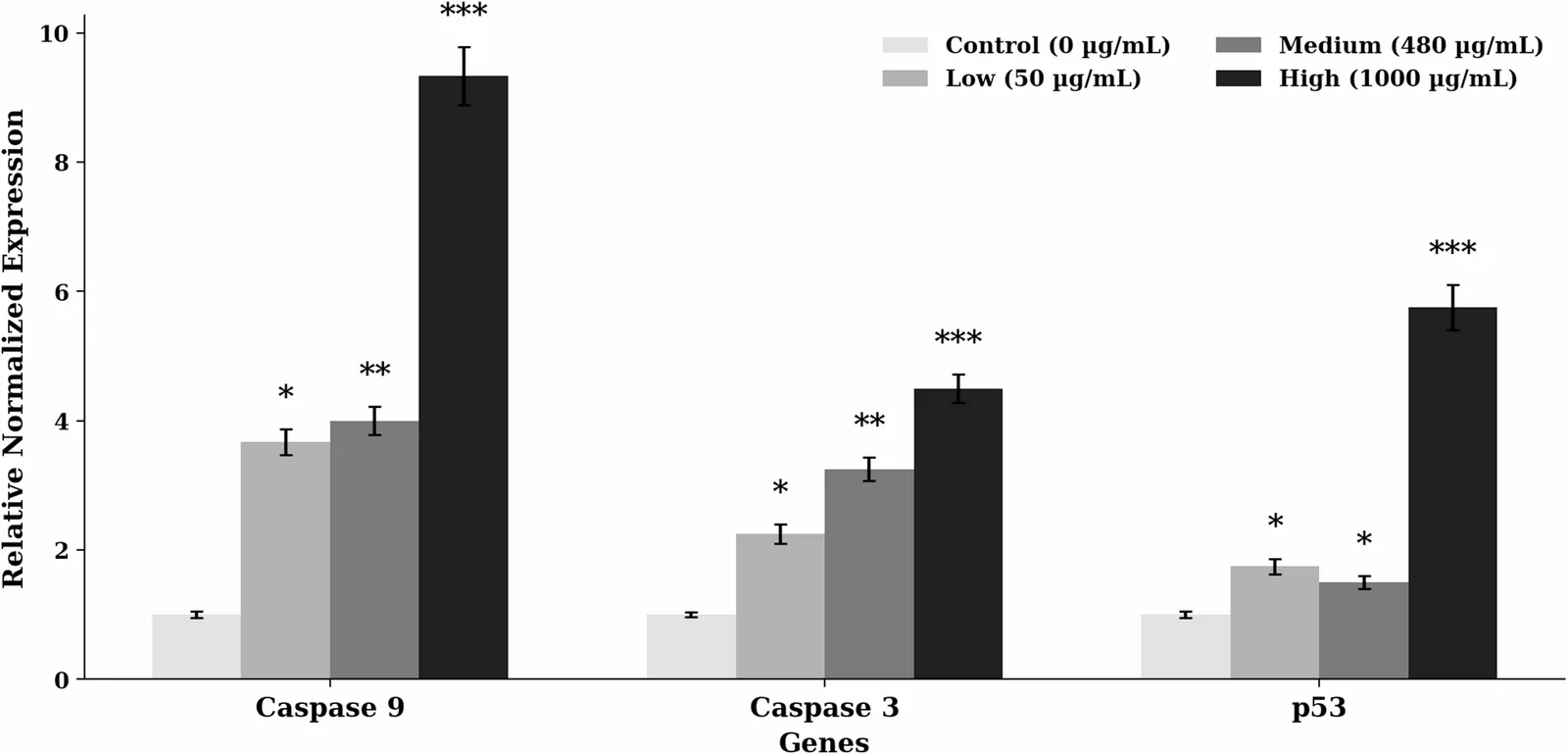
Relative expression of Caspase-9, Caspase-3 and P53 in control and treated groups (mean ± SD, *p < 0.05, **p < 0.01, ***p < 0.001).

Quantitative real-time PCR (qRT-PCR) analysis was performed to evaluate the transcriptional response of key apoptotic genes in AGS gastric carcinoma cells following treatment with biogenic ZnO nanoparticles synthesized from *Artemisia sieberi* (Fig. 6). A dose-dependent upregulation of Caspase-9, Caspase-3, and p53 was observed. At the highest concentration (1000 µg/mL), expression levels increased significantly compared to the control: Caspase-9 (~ 9.2-fold), Caspase-3 (~ 5.1-fold), and p53 (~ 5.7-fold) (*p* < 0.001). It should be noted that protein-level confirmation (e.g., Western blot) was not performed, and anti-apoptotic genes such as Bcl-2 and the Bax/Bcl-2 ratio were not assessed. Additionally, the housekeeping gene used for normalization was not specified, which may affect quantitative comparisons. These results demonstrate transcriptional activation of intrinsic apoptotic pathways in AGS cells, but mechanistic insight regarding the selectivity of ZnO NPs against AGS versus HEK-293 cells remains to be further investigated.

## Discussion

The comprehensive physicochemical characterization confirms the biogenic synthesis of zinc oxide (ZnO) nanoparticles using *Artemisia sieberi*, with findings that are highly consistent with and expand upon a growing body of literature in nanomedicine. FE-SEM and DLS analyses revealed nanoparticles with a predominantly spherical to polyhedral morphology and a narrow size distribution. This specific morphology is a common hallmark of plant-mediated synthesis, reflecting the intricate nucleation and growth processes influenced by the extract’s phytochemicals^20^. The observed agglomeration, a natural phenomenon for metal oxide nanoparticles due to their high surface energy, is a critical factor that can modulate surface reactivity and interactions with biological systems^21^. **A**gglomeration could also influence effective cellular uptake and cytotoxicity, potentially affecting the observed therapeutic outcomes. Furthermore, the elemental purity and high crystalline phase integrity, definitively confirmed by EDS and XRD, underscore the efficacy of this green synthesis approach in yielding high-quality, reproducible nanomaterials^22^.

The therapeutic promise of these nanoparticles is highlighted by their selective cytotoxicity against gastric carcinoma cells while demonstrating low toxicity toward normal cells. While the observed selectivity index (~ 2.6-fold) suggests preferential activity, this modest selectivity window should be interpreted cautiously as a limitation, and higher concentrations may affect normal cells. This preferential action is likely driven by the increased intracellular generation of reactive oxygen species (ROS) in cancer cells, which exploits their already elevated oxidative stress levels and compromised antioxidant defenses^23^. The rapid proliferation and altered membrane dynamics of cancer cells also facilitate a greater uptake of nanoparticles through enhanced endocytosis, further contributing to their differential susceptibility^24^. However, the precise molecular mechanisms underlying selective uptake and cytotoxicity require further investigation, including detailed studies on nanoparticle internalization and ROS measurements. These mechanisms are well-supported by studies showing that ZnO nanoparticles selectively induce cell death in various other cancer types, including breast and colon carcinomas^25^.

The mode of action was definitively identified as apoptosis, a highly desirable outcome in anticancer therapy due to its ability to circumvent the inflammatory response associated with necrosis. DAPI staining and flow cytometry provided compelling evidence of this programmed cell death, revealing key apoptotic hallmarks such as nuclear condensation and a significant increase in the apoptotic cell population following treatment^26^. While these data are supportive, protein-level confirmation of apoptotic markers (e.g., Caspase-3, Caspase-9, Bax/Bcl-2 ratio) and complementary mitochondrial assays would strengthen mechanistic conclusions.

Our molecular investigations provided a crucial mechanistic framework, confirming the activation of the intrinsic mitochondrial apoptotic pathway. The nanoparticles trigger a dose-dependent upregulation of p53, a pivotal tumor suppressor that acts as a cellular stress sensor. Upon activation by nanoparticle-mediated ROS production, p53 promotes the transcription of pro-apoptotic genes, ultimately inducing mitochondrial outer membrane permeabilization (MOMP) and the release of cytochrome c^27^. This cascade leads to the formation of the apoptosome and the subsequent activation of initiator Caspase-9, which then cleaves and activates executioner Caspase-3, leading to systematic cellular dismantling^28^. Again, protein-level validation is needed to fully confirm this pathway. This well-documented cascade is a critical therapeutic target in nanoparticle-mediated cytotoxicity^29^.

In addition to inducing apoptosis, the nanoparticles significantly inhibited cell migration, as shown by the wound healing assay. However, these results may be confounded by cytotoxicity, and additional assays are required to distinguish true anti-migratory effects from proliferation inhibition. Since metastasis is a major obstacle in cancer treatment, this anti-migratory effect is of immense clinical relevance^30^. The suppression of cell motility is likely mediated by the nanoparticles’ interference with key signaling pathways that regulate cytoskeletal dynamics and cell adhesion, notably the PI3K/Akt and MAPK/ERK pathways^31^. These proposed mechanisms are speculative in the current study and should be investigated in future work. The inhibition of these pathways, which are often hyperactive in cancer, leads to impaired migration and reduced metastatic potential. Other studies also point to the involvement of the NF-κB pathway, further emphasizing the multifaceted impact of these nanoparticles on complex cancer signaling networks^32^.

The green synthesis approach using *Artemisia sieberi* not only ensures a sustainable production method but also likely enhances the nanoparticles’ therapeutic efficacy. Phytochemicals present in the extract act as both capping and stabilizing agents, and may provide synergistic pro-oxidant or antioxidant effects; however, direct evidence comparing biogenic and chemically synthesized ZnO nanoparticles is required to confirm this contribution. These phytochemicals may modulate intracellular signaling cascades, thereby augmenting the observed apoptotic and anti-migratory responses^33^.

Overall, this study elucidates a comprehensive mechanism for the therapeutic action of biogenic ZnO nanoparticles. They act as multifunctional agents by inducing selective apoptosis via the p53-mediated mitochondrial pathway while concurrently suppressing migration through the inhibition of crucial signaling cascades. However, limitations such as modest selectivity, high concentrations required for activity, nanoparticle aggregation, and lack of in vivo validation should be acknowledged. This dual mode of action addresses two critical hallmarks of cancer progression—uncontrolled proliferation and metastasis—and underscores the proof-of-concept potential of plant-mediated ZnO nanoparticles for gastric cancer treatment^34^. Future research should focus on in vivo studies, detailed protein-level mechanistic validation, and exploration of combinatorial effects with existing chemotherapeutics to optimize clinical application.

Although this study provides comprehensive evidence for the selective cytotoxic and anti-migratory effects of *Artemisia sieberi*-mediated ZnO nanoparticles, certain mechanistic and comparative analyses were not conducted. Specifically, advanced physicochemical characterizations such as UV–Vi’s spectroscopy, Fourier Transform Infrared (FTIR) spectroscopy, and zeta potential measurements, as well as direct assessment of ROS generation and mitochondrial membrane potential changes, were not performed. Protein-level validation of apoptotic markers (cleaved Caspase-3, Caspase-9, and p53) and evaluation of anti-apoptotic genes (e.g., Bcl-2) with calculation of the Bax/Bcl-2 ratio was also not carried out. Furthermore, comparative studies using chemically synthesized ZnO nanoparticles without plant extracts are necessary to distinguish the intrinsic effects of ZnO from the contributions of phytochemicals. These limitations identify key areas for future research to fully elucidate molecular mechanisms and optimize the therapeutic potential of biogenic ZnO nanoparticles.

## Conclusion

The present study demonstrates that biogenic zinc oxide (ZnO) nanoparticles synthesized using *Artemisia sieberi* exhibit favorable physicochemical properties, including nanoscale dimensions, high purity, and a single-phase crystalline structure. These nanoparticles show selective cytotoxicity against AGS gastric cancer cells in vitro, primarily through apoptosis mediated by the intrinsic mitochondrial pathway, as indicated by morphological changes, flow cytometry, and upregulation of key apoptotic genes such as p53, Caspase-9, and Caspase-3. The nanoparticles also reduce cancer cell migration, highlighting potential anti-metastatic effects. Selective activity toward AGS cells relative to HEK cells suggests a degree of specificity. Overall, these findings provide a proof-of-concept in vitro framework for the therapeutic potential of *Artemisia sieberi*-mediated ZnO nanoparticles, supporting further studies to explore their mechanisms of action and potential applications in gastric cancer treatment.

## Data Availability

Data supporting the findings of this study are available upon reasonable request from the corresponding author.

## Declarations

### Artificial intelligence utilization for article writing

Artificial intelligence (AI) has not been used for writing this article.
